# Distinct brain pathways link depressive symptoms due to respiratory dysfunction and reduced physical activity in patients with chronic obstructive pulmonary disease: A single-center observational pilot study

**DOI:** 10.1371/journal.pone.0340067

**Published:** 2026-01-28

**Authors:** Shunsuke Sakakura, Motoyasu Honma, Yuri Masaoka, Ryo Manabe, Kentaro Okuda, Masaki Yoshida, Akira Yoshikawa, Misako Matsui, Daiki Shoji, Miku Kosuge, Shota Kosuge, Kenta Miyo, Masahiro Ida, Fumihiro Yamaguchi, Takuya Yokoe, Masahiko Izumizaki

**Affiliations:** 1 Department of Physiology, Showa Medical University School of Medicine, Tokyo, Japan; 2 Department of Respiratory Medicine, Showa Medical University Fujigaoka Hospital, Yokohama, Japan; 3 Department of Medicine, Division of Respiratory Medicine and Allergology, Showa Medical University School of Medicine, Tokyo, Japan; 4 Department of Medicine, Tokyo Metropolitan Ebara Hospital, Tokyo, Japan; 5 Home Care Support Clinic Kaede no Kaze Kawasaki Chuo, Kawasaki, Japan; 6 Department of Orthoptics and Visual Sciences, International University of Health and Welfare, Tokyo, Japan; 7 Division of Health Science Education, Showa Medical University School of Nursing and Rehabilitation Sciences, Yokohama, Japan; 8 Department of Neurology, Showa Medical University School of Medicine, Tokyo, Japan; 9 Department of Radiology, Stroke Center, Tokyo Metropolitan Ebara Hospital, Tokyo, Japan; 10 Department of Radiology, National Hospital Organization Mito Medical Center, Ibaraki, Japan; RIKEN CBS: RIKEN Noshinkei Kagaku Kenkyu Center, JAPAN

## Abstract

Mental health issues, such as depression, are increasingly recognized as critical comorbidities in chronic obstructive pulmonary disease (COPD). While analyses of individual domains or pairwise relationships among airflow obstruction severity, brain morphological features, and mental health conditions in COPD have provided valuable insights into their direct associations, a more integrative approach may offer additional mechanistic understanding. This study aimed to clarify potential mediators in multiple brain regions between airflow obstruction and depressive tendencies in patients with COPD. We analyzed 19 patients with COPD and 23 age-matched healthy controls. Path analysis was used to evaluate the relationships among respiratory indices, regional brain volumes, and depressive symptoms. The path analysis revealed two potential pathways to depressive symptoms in the COPD group. One was a pulmonary pathway, involving forced expiratory volume in one second (%FEV_1_) and the genu of the corpus callosum (β = 0.503, *p* < 0.05) leading to depressive symptoms (β = 0.532, *p* < 0.05). The other was a behavioral pathway, in which physical activity (β = 0.444, *p* < 0.05) affected the right postcentral gyrus (β = −0.286, *p* < 0.05) and, in turn, depressive symptoms. No significant group differences were observed in other examined regions, including the posterior cingulate, hippocampus, hypothalamus, insula, or other corpus callosum subregions, nor in depression or anxiety scores. The model demonstrated a good fit in the COPD group (GFI = 0.876), whereas in controls, the model showed poor fit and no significant relationships (GFI = 0.784). These preliminary findings suggest potential brain pathways linking airflow obstruction, physical activity, and depressive symptoms in COPD, which warrant confirmation in larger, longitudinal studies.

## Introduction

Chronic obstructive pulmonary disease (COPD) is a heterogeneous lung condition characterized by persistent, often progressive airflow obstruction resulting from abnormalities in both the airways and alveoli (Global Strategy for the Prevention, Diagnosis and Management of COPD: 2025 Report (GOLD)) [1]. The disease is primarily triggered by chronic exposure to toxic substances, particularly cigarette smoking [2] and air pollutants [3], and presents with respiratory symptoms such as chronic dyspnea and productive cough.

COPD is a systemic disease with inflammatory features that extend beyond the lungs. It is commonly associated with comorbidities, including ischemic heart disease, heart failure, osteoporosis, normocytic anemia, lung cancer, depression, and diabetes, all of which contribute to poorer patient outcomes [4]. Although often underrecognized, psychiatric disorders, which include depression and anxiety, are highly prevalent among patients with COPD [5]. A large matched cohort study reported that patients with COPD have a 42% higher risk of developing incident depression compared to matched controls without COPD, with the risk being particularly elevated in those experiencing more severe dyspnea [6]. These mental health conditions are significantly associated with worsened health status, increased risk of exacerbations, and higher rates of emergency hospital admissions in patients with COPD [7]. According to the GOLD Report [1], depression is among the three most frequently overlooked comorbidities in COPD, along with heart failure and lung cancer, underscoring the need for systematic mental health screening in comprehensive COPD management [1] (p.124).

While COPD management incorporates comprehensive assessment tools such as the GOLD ABE classification [1], forced expiratory volume in one second (FEV_1_) remains a fundamental physiological marker used to objectively quantify the severity of airflow obstruction [1] (p.40). Neuroimaging studies have demonstrated significant associations between the severity of airflow obstruction and structural changes in the brain. For example, one study demonstrated that greater airflow obstruction is linked to widespread brain alterations [8], including reductions in cerebral white matter volume and microstructural abnormalities associated with poorer mood states. These findings suggest that anxiety and depression in COPD may occur secondary to white matter damage. Another investigation found that patients with moderate airflow obstruction, as measured by FEV_1_, exhibited reduced gray matter density in frontal regions and compromised white matter integrity, evidenced by increased diffusivity in major tracts, particularly the corpus callosum and corona radiata, with changes becoming more pronounced in cases of severe obstruction [9]. A case-control study reported reduced gray matter in several key regions, including the midcingulate cortex, anterior cingulate cortex, hippocampus, and amygdala, in patients with COPD compared to healthy controls. Notably, a mediation study revealed a significant indirect effect: patients with longer disease duration exhibited a greater fear of physical activity, which was associated with more pronounced volume reduction in the anterior cingulate cortex [10]. This mediation approach highlights how COPD may affect mental health through structural brain changes, rather than via isolated correlations.

Although FEV_1_ alone does not fully capture the complex effects of COPD on quality of life, its robust association with brain structure offers an opportunity to investigate the neurobiological pathways linking COPD pathophysiology to psychological symptoms. Building on these findings, we hypothesize that structural alterations in specific brain regions mediate the relationship between the airflow obstruction severity and depression or anxiety symptoms in patients with COPD. To test this hypothesis, we conducted a path analysis focusing on regions of interest (ROI) previously implicated in COPD-related brain changes, including the cingulate cortex, hippocampus, amygdala, postcentral gyrus, insula cortex, and corpus callosum [11–15]. These regions are crucial for emotional processing and stress regulation, and their structural abnormalities may mediate the association between the airflow limitation and depression or anxiety symptoms.

## Materials and methods

### Ethics statement

This study was approved by the Ethics Committee of Tokyo Metropolitan Ebara Hospital, Tokyo Metropolitan Hospital Organization (trial identifier number: 2811) and conducted in accordance with the Declaration of Helsinki. The study protocol was prospectively registered in the University Hospital Medical Information Network (UMIN) registry. (UMIN-CTR; trial ID: UMIN000054768). Written informed consent was obtained from all participants. The authors had access to identifying participant information during and after data collection.

### Participants

**Fig 1:**
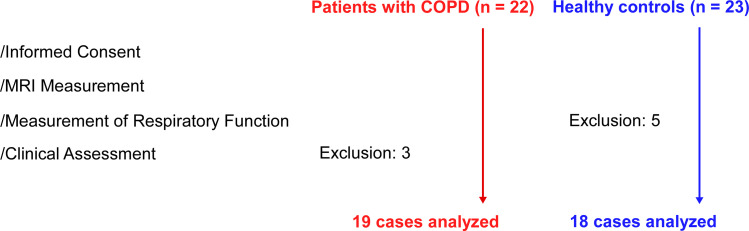
Patient flow diagram. Three patients with COPD did not complete the questionnaires for the clinical assessments, and 5 healthy controls did not complete the measurement of respiratory function. The 19 patients and 18 healthy controls underwent MRI acquisition, and respiratory function testing, and clinical assessments. Background factors (e.g., BMI, smoking history) were obtained prior to the clinical evaluations.

This single-center cohort study was conducted at Tokyo Metropolitan Ebara Hospital. Between 1 November 2016–12 January 2017, 22 patients with COPD were recruited. Of these, 19 patients (18 males) were included in the analysis; three were excluded due to missing data (no completed questionnaires) (Fig 1). A total of 23 age-matched healthy controls (16 males) were selected from a previously published study [16]; these individuals underwent structural magnetic resonance imaging (MRI) using the same MRI acquisition protocol, image processing, and volume segmentations. Five control subjects were excluded from the path analysis due to missing respiratory data.

### Measurement of respiratory function

All participants underwent standard spirometry to assess parameters, including forced vital capacity (FVC) and FEV_1_. The percent predicted FEV_1_ (%FEV_1_) was calculated as the ratio of measured FEV_1_ to the predicted FEV_1_, based on age, sex, and height. Spirometry was performed without additional odilator administration; however, patients with COPD remained on their prescribed medications. Healthy controls followed the same spirometry protocol, also without bronchodilator use.

### Assessments

Data on age, body mass index (BMI), and smoking history of the participants were collected. Physical activity was evaluated using the International Physical Activity Questionnaire (IPAQ), which measures the number of days and duration of moderate-and high-intensity physical activity during a week [17]. COPD severity and disease duration were recorded, and the COPD Assessment Test (CAT) was used to evaluate disease impact [18]. Anxiety and depression were assessed using the Hospital Anxiety and Depression Scale (HADS) [19], which comprises 14 items (7 for depression and 7 for anxiety). Each item is scored from 0 to 3, with subscale scores calculated as the sum of the item scores; higher scores indicate poorer mental health.

### MRI acquisition

Structural MRI scans were acquired at Ebara Hospital using a 3.0-Tesla Siemens Trio system (Erlangen, Germany) with standard head coil settings. T1-weighted anatomical images were acquired using the following parameters: repetition time = 2250 ms, echo time = 3.06 ms, flip angle = 9°, inversion time = 1000 ms, field of view = 256 × 256 mm, matrix size = 256 × 256, and voxel size = 1 × 1 × 1 mm. High-resolution anatomical imaging was performed using a magnetization-prepared rapid gradient echo (MPRAGE) sequence.

### Image processing

Image processing was conducted using FreeSurfer version 6.0 (http://surfer.nmr.mgh.harvard.edu) [20]. The pipeline included motion correction, removal of non-brain tissue, intensity normalization, affine registration to Montreal Neurological Institute (MNI) space, and Talairach transformation. Volumetric segmentation [21], cortical surface reconstruction [22–24], and parcellation [25–27] were performed automatically using the recon-all script. Gray matter volumes were defined based on the Desikan-Killiany brain atlas [26], and all boundaries were visually verified by two trained neurologists using FreeView following affine registration.

Each regional brain volume was adjusted relative to total intracranial volume (ICV) to minimize the effect of head-size differences across individuals [28]. The ratio-corrected volumes were calculated as the ratio of regional brain volume to the ICV. The residual method applied the following correction (1):


CV=V−S (ICV−ICVmean) (1)

CV is the corrected regional brain volume, V is the original volume, S is the slope of the linear regression of V on ICV, ICV is the intracranial volume for a particular participant, and ICVmean is the mean ICV across participants.

### Statistical analysis

Unpaired t-test were used to assess differences between the COPD and control groups in age, BMI, educational history, smoking history, %FEV_1_, and volumes of the left and right posterior cingulate, hippocampus, hypothalamus, insula, postcentral gyrus, and body/ampulla/genu of the corpus callosum, as well as depression, and anxiety scores. All tests were two-tailed. Quantitative results are summarized as the mean together with the standard error (SEM). SPSS version 26 was utilized for the analysis. Path analysis was conducted using AMOS version 27.0. Three sets variables were included: (1) respiratory function indices (activity level, smoking history, disease duration, CAT, and %FEV_1_), (2) regional brain volumes (left/right of posterior cingulate, hippocampus, hypothalamus, insula, postcentral, and body/ampulla/genu of corpus callosum), and (3) psychological measures (depression and anxiety). Path analysis was performed separately for each group. The goodness of fit index (GFI) was calculated to assess model fit.

## Results

The basic characteristics of the participants, respiratory function measures, regional brain volume, and depression/anxiety scores are summarized in Table 1. Unpaired t-test revealed no significant differences between the groups in age, BMI, years of education, or physical activity. However, a significant group difference was observed in smoking history (t_35_ = 3.3324, *p* = 0.002). The %FEV_1_ was also significantly lower in COPD group compared to controls (t_35_ = 4.622, *p* < 0.0001). No significant group differences were found in volumes of the posterior cingulate, hippocampus, hypothalamus, and insula (all *p* > 0.300). Similarly, the body, ampulla, and genu of the corpus callosum did not differ significantly between groups (all *p* > 0.05). Depression and anxiety scores on the HADS also showed no significant differences between the two groups (*p* > 0.600).

**Table 1:** Participants’ characteristics: Demographics, clinical, neuroimaging, and psychiatry measures.

		Patients		Controls		
		Average	S.D.	Average	S.D.	*p* value
Age		73.05	5.85	74.46	4.93	0.397
BMI		23.21	3.76	23.31	2.00	0.907
Education history (year)		12.68	1.89	14.17	2.72	0.062
Activity		207	216	132	117	0.203
Smoking history		1.63	0.50	2.40	0.75	0.007
Duration of disease (year)		4.29	4.81	–	–	–
CAT		15.05	6.22	–	–	–
%FEV_1_ (%)		66.69	20.97	95.19	16.07	< 0.0001
Posterior cingulate (mm^3^)						
	Left	3103	376	3205	369	0.383
	Right	2950	525	2914	373	0.796
Hippocampus (mm^3^)						
	Left	3560	295	3500	367	0.565
	Right	3687	293	3710	458	0.853
Amygdala (mm^3^)						
	Left	1317	185	1371	217	0.407
	Right	1537	147	1518	229	0.767
Hypothalamus (mm^3^)						
	Left	5843	449	5783	445	0.669
	Right	5874	409	5758	415	0.373
Insula (mm^3^)						
	Left	5570	481	5538	506	0.838
	Right	5839	413	5811	386	0.823
Postcentral (mm^3^)						
	Left	9309	1089	9694	1133	0.300
	Right	8766	1325	9264	1254	0.249
Corpus callosum (mm^3^)						
	Body	443	59	414	133	0.839
	Ampulla	1013	103	891	159	0.050
	Genu	845	135	773	123	0.139
Depression		3.00	2.89	3.39	2.62	0.671
Anxiety		3.58	2.29	3.28	2.26	0.708

BMI: Body Mass Index, CAT: COPD assessment test, %FEV_1_: percent predicted Forced Expiratory Volume in one second. Smoking history was examined using a 3-point Likert method 1 = current smoker, 2 = former smoker in the past, 3 = never smoker.

**Fig 2:**
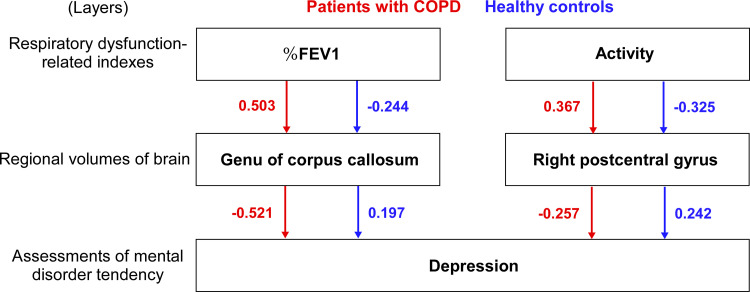
Path analysis model showing two possible pathways to depressive symptoms. One pathway involves %FEV_1_, the genu of the corpus callosum, and depressive symptoms. The other pathway involves physical activity, the right postcentral gyrus, and depressive symptoms. Red arrows and values indicate standardized regression weights for the COPD patient group, while blue arrows and values represent those for the healthy control group.

For the path analysis, we constructed a three-layer model: (1) respiratory function-related indices, (2) regional volumes, and (3) assessments of depressive and anxiety tendencies. All full models, including all paths between these layers, were tested and then refined by removing non-significant paths (Figs 2 and S1). Path analyses were conducted separately for each group. In the COPD group, the final model demonstrated the best goodness-of-fit (*X*^2^(6) = 5.733, *p* = 0.454, GFI = 0.904, Bollen-Stine bootstrap *p* = 0.465). The endogenous variables were connected via single-headed arrows, representing standardized regression weights (β). In this model, the %FEV_1_ had a direct effect on the genu of the corpus callosum (β = 0.503, *p* < 0.01), and physical activity directly impacted the right postcentral gyrus (β = 0.367, *p* < 0.05). Both the genu of the corpus callosum (β = −0.521, *p* < 0.05) and right postcentral gyrus (β = −0.257, *p* < 0.05) showed a direct effect on depression score. In contrast, the model demonstrated poor fit in the healthy control group (*X*^2^(6) = 6.517, *p* = 0.368, GFI = 0.784, Bollen-Stine bootstrap *p* = 0.515).

## Discussion

The path analysis suggests a model consisting of two distinct pathways leading to depressive symptoms in the COPD group. The first is a pulmonary pathway, in which lower %FEV_1_ is associated with a smaller volume of the genu of the corpus callosum, which in turn is linked to greater depressive symptoms. The second is a behavioral pathway, in which reduced physical activity is associated with a smaller right postcentral gyrus, which likewise shows a significant relationship with depressive symptoms. Notably, the same model demonstrated a poor goodness-of-fit in the healthy control group, suggesting that these relationships are specific to the COPD population.

Although the mean depression scores did not differ significantly between the COPD and control groups, we modeled depressive symptoms as the primary outcome to examine within-group associations among respiratory function, brain structure, and mood. This analytical focus was grounded in the rationale that subthreshold depressive symptoms—even when clinical group differences are absent—may nonetheless reflect meaningful neural mechanisms and vulnerability to mood disturbances. Previous studies have similarly reported that such subtle affective variations can be linked to structural and functional brain alterations in chronic medical conditions, supporting the importance of exploring these relationships in COPD. While group-level differences in depressive symptoms or regional brain volumes were not observed, the path analysis was specifically designed to identify latent brain–behavior relationships within the COPD group. This approach provides mechanistic insight into how subclinical mood variations may arise from underlying physiological and neural alterations, even in the absence of overt clinical differences. Modeling these within-group pathways allows for the detection of continuous, disease-related changes and offers a framework for understanding the early or subthreshold manifestations of neuropsychiatric symptoms in COPD. Taken together, these findings suggest that the risk of depression in COPD may be closely linked to structural alterations in the corpus callosum and postcentral gyrus, both of which appear to be affected by airflow obstruction and physical inactivity.

The relationship between COPD and structural brain alterations has been well documented. Prior studies have provided crucial evidence of progressive structural impairments in both gray matter and white matter as airflow obstruction worsens, with significant white matter changes in the corpus callosum and cingulum correlating with cognitive deficits [9]. Further research reported that axial diffusivity reductions were most pronounced in the corpus callosum, corticospinal tracts, and midbrain among patients with COPD [29]. Our findings extend these observations by demonstrating a specific association between reduced %FEV_1_ and genu atrophy, indicating that this region may be particularly vulnerable. In addition, regular physical activity has been shown to exert a favorable effect on various cerebral regions. One study comparing athletes and non-athletes found that athletes had greater volumes in the right frontal lobe, left corticospinal tract, and right postcentral gyrus [30]. Another study comparing individuals in physically demanding versus less physically demanding occupations reported greater cortical thickness in the right postcentral gyrus among the former group [31]. These findings suggest a strong association between activity and postcentral gyrus volume, aligning with our current results.

The mechanistic link between respiratory dysfunction and brain structural changes likely involves multiple pathways. A previous study suggested that white matter abnormalities in patients with COPD are largely independent of smoking history and cerebrovascular comorbidities, implicating hypoxemia and systemic inflammation as primary drivers [32]. This is consistent with findings from patients with obstructive sleep apnea, a condition marked by intermittent hypoxia, who also show structural deformities in the genu and rostral body of the corpus callosum [33]. Another study reported that improved working memory after CPAP (continuous positive airway pressure) therapy was significantly associated with decreased deactivation in the posterior cingulate and postcentral gyrus, while increased sleepiness was linked to activation in the left and right medial frontal gyrus [34]. Our findings support these results by demonstrating that structural alterations in the corpus callosum and postcentral gyrus are integral to COPD pathophysiology.

The clinical relevance of corpus callosum damage, particularly in the genu, extends beyond COPD. Structural disruption in this region has been linked to cognitive and emotional impairments, with the genu being critical for interhemispheric communication and higher-order cognitive processes [35]. In bipolar disorder, reduced fractional anisotropy in the corpus callosum has been associated with executive dysfunction and impaired emotional regulation [36], while disturbances in the genu have been implicated in alexithymia [37]. Case reports of corpus callosum infarction involving the genu have demonstrated associations with severe memory deficits and impaired emotional processing [38], consistent with our findings on the relationship between genu integrity and depressive symptoms. Additionally, the postcentral gyrus has been strongly associated with psychiatric disorders, including depression. A recent study reported reduced structural and functional connectivity in the parietal lobe among patients with major depressive disorder (MDD) [39]. Another investigation utilizing magnetization transfer imaging found increased gray matter volume in the right postcentral gyrus in patients with MDD [40]. These findings highlight the postcentral gyrus as a region implicated in depression symptoms, further supporting our model.

Recent insights into the lung–brain axis further illuminate these connections. A study showed that chronic pulmonary diseases, including COPD, are associated with neuroinflammatory and oxidative stress, which disproportionately affect white matter integrity [41]. Consistent with this notion, a recent review highlighted that COPD-related neuroinflammatory processes contribute to both structural brain alterations and comorbid depression, underscoring the bidirectional interaction between pulmonary and neuropsychiatric health [42]. The present study delineates a specific mechanistic link within this broader framework: reductions in %FEV_1_ and physical activity lead to atrophy in the genu of the corpus callosum and postcentral gyrus, which in turn are associated with depressive tendencies. This indicates that these brain regions are not only sites of structural damage but also critical mediators of broader network dysfunction in COPD.

This study has limitations. First, the relatively small sample size (19 patients with COPD and 23 healthy controls) may limit the robustness of the path analysis and increase the risk of overfitting. The findings should therefore be interpreted as exploratory and preliminary. While the model demonstrated an acceptable goodness of fit, the relationships identified may not be stable or fully generalizable to broader COPD populations. Small samples can lead to biased estimates and unstable structural equation model solutions, highlighting the need for replication in larger, multicenter cohorts [43]. Second, although our results revealed a significant association between right postcentral gyrus volume and depressive symptoms, the directionality and biological meaning of this relationship remain to be clarified. The postcentral gyrus is implicated in somatosensory–affective integration, and its observed structural alterations may represent compensatory or secondary changes rather than a primary cause of depressive symptoms. Previous studies have demonstrated that cortical thinning is associated with frailty and physical inactivity [44], suggesting that the changes observed in the corpus callosum and postcentral gyrus in our study represent part of a broader neuropsychiatric phenotype in COPD. The involvement of somatosensory cortices in affective processing has been highlighted in recent neuroimaging reviews [45,46], supporting the notion that sensory–affective coupling may contribute to mood dysregulation. Third, potential confounding factors should be considered when interpreting the present findings. Although participants with major psychiatric or neurological disorders were excluded, we did not fully control for the effects of comorbidities, psychotropic medication use, or smoking burden. In particular, cumulative smoking exposure (pack-years) and systemic medications such as corticosteroids could influence both brain structure and mood regulation. Antidepressants and other psychotropic drugs are known to modulate emotional processing and neural plasticity [47,48], which may have contributed to variability in the observed associations. Fourth, potential selection bias may have influenced our findings because three patients with COPD were excluded from the analysis due to missing questionnaire. These excluded participants might have differed systematically in disease severity, mental health status, or data quality, which could limit the representativeness of the final sample. Furthermore, the potential effects of antidepressant and anxiolytic medications should be acknowledged. Although we recorded medication use, the small sample size did not allow for separate analysis of medicated versus non-medicated participants. These drugs can influence both mood regulation and neural plasticity, potentially confounding the observed associations between brain structure and depressive symptoms. Fifth, smoking exposure was re-evaluated using pack-years rather than a categorical Likert scale to more precisely quantify cumulative burden. This approach allowed a finer discrimination of lifetime exposure, although statistical power remained limited to detect its independent effects on brain structure and mood. Chronic smoking has been associated with widespread structural and cognitive alterations [49,50], and its contribution to neural vulnerability in COPD warrants further investigation. Sixth, the cross-sectional design, limited region-of-interest approach, and lack of functional neuroimaging constrain causal inference and comprehensive network-level interpretation. Structural MRI alone cannot fully capture brain network dynamics or functional coupling relevant to mood regulation [51,52]. Larger, longitudinal, multimodal studies that incorporate both structural and functional measures—and use objective indicators of physical activity rather than self-reported data [53]—are warranted to validate and extend the present findings. Finally, this study employed a single-center design, which may limit the generalizability of the findings. The characteristics of patients treated at our institution, as well as institution-specific clinical practices, may not fully represent those of broader COPD populations. Future multi-center studies with larger and more diverse samples are needed to validate these results.

## Conclusions

This pilot study examined the relationships among the severity of airflow obstruction, physical activity, brain morphology, and mental health in patients with COPD using path analysis. Two distinct pathways, pulmonary and behavioral, were identified as potential mechanisms contributing to depressive symptoms. Specifically, reduced %FEV_1_ and physical activity were each associated with atrophy in the genu of the corpus callosum and the postcentral gyrus, respectively, which in turn correlated with higher depressive symptoms. The model demonstrated a good fit, suggesting that these two brain regions may help explain the link between respiratory dysfunction and depression in COPD. Future studies should incorporate both structural and functional neuroimaging techniques to more precisely investigate how COPD-related systemic effects disrupt neural connectivity. Advanced imaging modalities, such as functional MRI and diffusion tensor imaging, could elucidate the relationship between localized structural changes and network-wide communication deficits. Additionally, therapeutic strategies aimed at reducing systemic inflammation and hypoxia may help mitigate brain damage and its psychological consequences in patients with COPD. Expanding the research scope to include related conditions, such as sleep disorders and adjustment disorders, may further enrich our understanding of the neuropsychiatric manifestations of COPD.

## Supporting information

S1 Fig Scatter plots illustrating the relationships among variables included in the path analysis. (PDF)
