# Supplementary material for: Distinct brain pathways link depressive symptoms due to respiratory dysfunction and reduced physical activity in patients with chronic obstructive pulmonary disease: A single-center observational pilot study
Source: PLoS One. 2026 Jan 28;21(1):e0340067. doi: 10.1371/journal.pone.0340067 (PMC12851480; doi:10.1371/journal.pone.0340067)
Supplement: S1 Fig — (PDF) [file pone.0340067.s001.pdf]

Supporting information

Distinct brain pathways link depressive symptoms due to respiratory dysfunction and reduced physical activity in patients with chronic obstructive pulmonary disease: A single-center observational pilot study

Shunsuke Sakakura, Motoyasu Honma, Yuri Masaoka, Ryo Manabe, Kentaro Okuda, Masaki Yoshida, Akira Yoshikawa, Misako Matsui, Daiki Shoji, Miku Kosuge, Shota Kosuge, Kenta Miyo, Masahiro Ida, Fumihiro Yamaguchi, Takuya Yokoe, Masahiko Izumizaki

S1 Fig

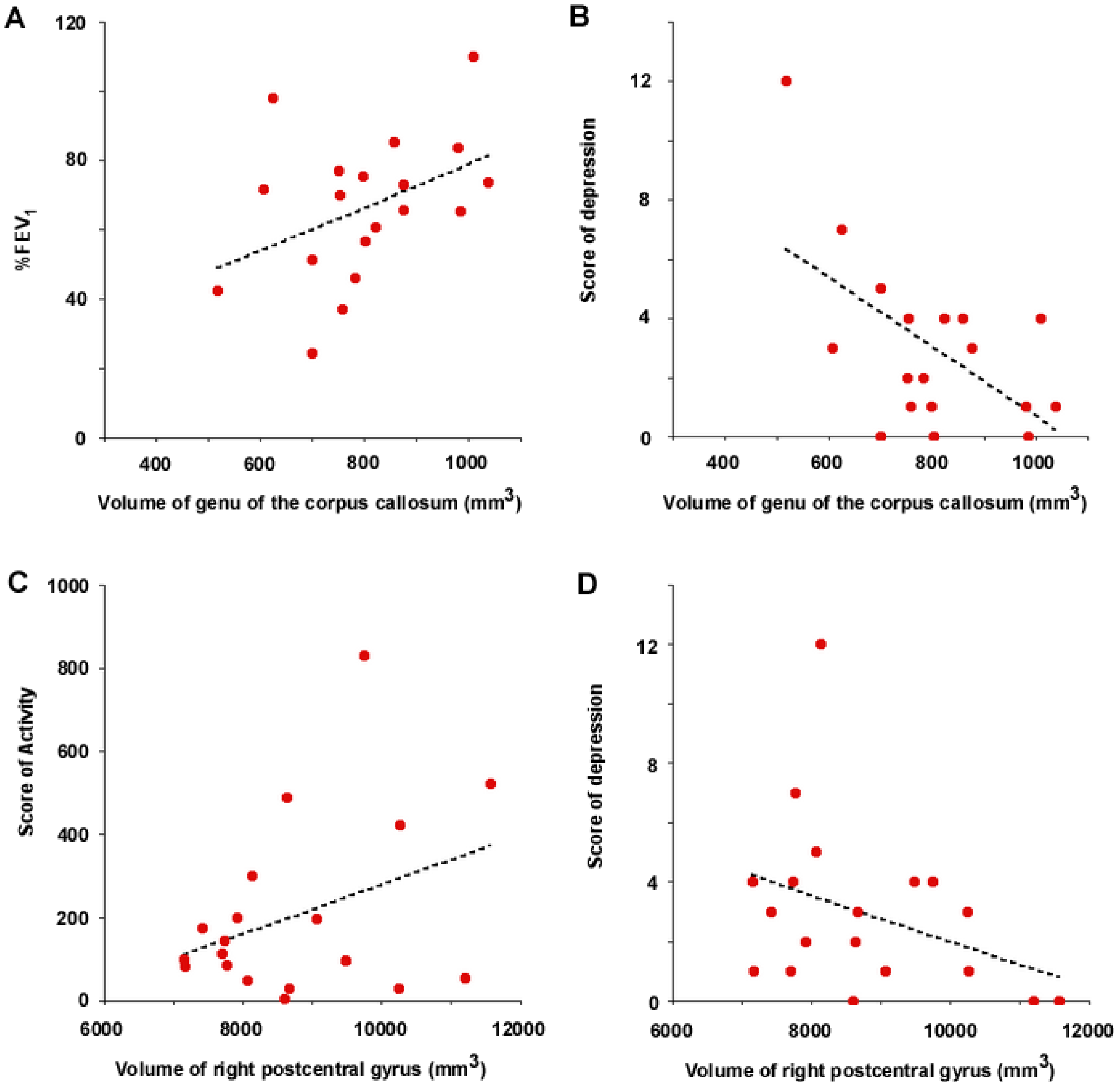

S1 Fig. Scatter plots illustrating the relationships among variables included in the path analysis. (A) Relationship between %FEV1 and the volume of the genu of the corpus callosum. (B) Relationship between depression score and the volume of the genu of the corpus callosum. (C) Relationship between activity score and the volume of the right postcentral gyrus. (D) Relationship between depression score and the volume of the right postcentral gyrus.
